# MiR-103 protects from recurrent spontaneous abortion via inhibiting STAT1 mediated M1 macrophage polarization

**DOI:** 10.7150/ijbs.46144

**Published:** 2020-05-25

**Authors:** Xiaoxiao Zhu, Haiping Liu, Zhen Zhang, Ran Wei, Xianbin Zhou, Zhaoxia Wang, Lin Zhao, Qiang Guo, Yunhong Zhang, Chu Chu, Li Wang, Xia Li

**Affiliations:** 1 Laboratory for Molecular Immunology, Institute of Basic Medicine, Shandong Provincial Hospital Affiliated to Shandong First Medical University, 18877 Jingshi Road, Jinan 250062, Shandong, China.; 2 Reproductive Medicine Center, The 960th Hospital of the PLA Joint Logistics Support Force, 25 Wuyingshan Road, Jinan 250031, Shandong, China.; 3 School of Medicine and Life Sciences, University of Jinan-Shandong Academy of Medical Sciences, 18877 Jingshi Road, Jinan 250062, Shandong, China.; 4 Department of Obstetrics and Gynecology, The First Affiliated Hospital of Shandong First Medical University, Shandong Provincial Qianfoshan Hospital, 16766 Jingshi Road, Jinan 250014, Shandong, China.

**Keywords:** recurrent spontaneous abortion, STAT1, miR-103, M1 macrophage, post-transcriptional regulation

## Abstract

Recurrent spontaneous abortion (RSA) is a common complication of early pregnancy. Excessive M1 macrophage was found to be involved in RSA, but the underlying mechanisms remains unclear. MicroRNAs play critical roles in RSA as well as the polarization of macrophages; however, the regulatory effect of miRNAs on M1 differentiation in RSA has not been fully investigated. In this study, miRNA microarray assay revealed that miR-103 was significantly decreased in RAW264.7-derived M1 macrophages upon IFNγ and LPS stimulation. Quantitative real-time polymerase chain reaction (qRT-PCR) analysis showed that in RSA patients, miR-103 expression was decreased substantially, and negatively correlated with that of STAT1. Moreover, down-regulation of miR-103 could sensitively discriminate RSA patients from normal pregnancies (NP) subjects. Experiments *in vitro* showed that overexpression of miR-103 suppressed M1 polarization by inhibiting STAT1/IRF1 signaling pathway and vice versa. miR-103 regulated STAT1 expression by direct binding to its 3'-UTR. Moreover, our *in vivo* study demonstrated that overexpressed miR-103 could reduce mice embryo resorption and M1 polarization effectively. Overall, the results suggested that decreased miR-103 was involved in RSA by increasing M1 macrophage polarization via promoting STAT1/IRF1 signaling pathway. miR-103 may be explored as a promising diagnostic marker and therapeutic target for RSA.

## Introduction

Recurrent spontaneous abortion (RSA) defined as two or more consecutive spontaneous abortions before 20 weeks of gestation 1-3, accounts for approximately 15% of clinically recognized pregnancies and affects approximately 5% of child-bearing age women heath around the world, according to the World Health Organization (WHO) estimate 4,5. Numerous studies have sought to determine the pathogenesis of RSA. However, except for the known pathogenic factors, including chromosomal abnormalities, endocrinological factors and anatomic abnormalities, the cause of approximately 50% RSA cases remains unexplained and further investigation is urgently needed 6. Macrophages, an important immunologic cell type, were originally described as classically activated macrophage (M1) characterized by high antigen presentation, high expression of pro-inflammatory cytokines and alternatively activated macrophage (M2) associated with an anti-inflammatory profile in response to environmental stimuli 7. The maternal-fetal interface is infiltrated by immune cells throughout pregnancy, and macrophages account for 20-30% of the total decidual leukocytes in the first trimester. Polarization balance of M1/M2 macrophage is necessary for a successful pregnancy, while the imbalance of M1/M2 during pregnancy is associated with spontaneous abortion or RSA 8. Studies demonstrated that the fraction of M1 macrophages in decidual of spontaneous abortions patients is significantly higher than that in normal pregnancies (NP). Macrophage activation towards a more M1 phenotype was associated with pregnancy loss 9. However, the factors and underlying mechanisms for triggering excessive M1 differentiation in RSA remains unclear.

Signal transducer and activator of transcription 1 (STAT1) is a prototypical member of the STAT family encoded by STAT1 gene located on human chromosome 2, which plays an important role in modulating macrophage polarization 10. It was found that activated STAT1 translocate into the nucleus and transduces extranuclear signals to the nucleus, thus activating the transcription of interferon regulatory factor-1 (IRF1) 11,12. IRF1, a transcriptional regulator, is only weakly expressed in resting macrophages, but can be strongly upregulated in M1 macrophage 13. Recent studies showed that IRF1 is a key effector protein in M1 polarization, while the knockout of IRF1 resulted in a decrease of M1 macrophage and an increase of M2 macrophages 13,14. However, it is not clear whether M1 elevation is related to STAT1/IRF1 pathway in patients with RSA.

miRNAs are endogenous short (21-24 nucleotide long) non-coding RNAs, which regulate target gene expression by binding to the 3' untranslated region (3'UTR) of mRNAs for degradation or translational repression 15,16. miRNAs are reported to be involved in various basic cellular processes, including cell differentiation, proliferation, migration, invasion and apoptosis 17,18. Recently, there is an increasing focus on the role of miRNAs in RSA 19-22. For example, it was found that the expression of miR-365 and miR-133a in villi of RSA patients was significantly higher than that in NP women 19,22. Moreover, miR-184 was reported to be up-regulated in the decidua of RSA patients to promote apoptosis and repress proliferation of trophoblast cells and induces early spontaneous abortion via targeting zinc finger matrin-type 3 (WIG1) 21. In addition, studies have demonstrated that miRNAs also play an essential role in macrophage differentiation 23-28. For instance, miR-181a can induces macrophage polarized to M2 phenotype by targeting kruppel like factor 6 (KLF6) and CCAAT enhancer binding protein alpha (C/EBPα) 27. Overexpression of let-7c in granulocyte-macrophage colony-stimulating factor (GM-CSF) treated bone marrow cells (M1 macrophages) diminishes M1 phenotypic expression, while promoting macrophage polarization toward to M2 phenotype 28. miR-99a could inhibit M1 macrophage phenotype by targeting tumor necrosis factor (TNF) 26. miR-130a was expressed at a higher level in M1 compared with M2 macrophages, which suppressed the polarization of macrophages to the M2 phenotype and enhanced M1 polarization by suppressing peroxisome proliferator-activated receptor (PPARγ) 29. Those studies suggested that miRNAs are involved in RSA as well as the polarization of macrophages. However, the regulatory effect of miRNAs on M1 differentiation in RSA has not been fully investigated.

In the present study, we investigated the regulatory role and implications of aberrant expression of miR-103 in RSA. We reported that miR-103 is down-regulated in RSA, and aggravate RSA by promoting M1 polarization via STAT1/IRF1 pathway. miR-103 could inhibit STAT1 expression by binding to its 3'UTR, and up-regulated miR-103 could alleviate RSA by suppressing M1 polarization via inhibiting STAT1/IRF1 pathway.

## Materials and Methods

### Patients

Between September 2017 to December 2018, 30 RSA patients admitted to the Reproductive Medicine Center of the 960th Hospital of the PLA Joint Logistics Support Force of Shandong Province and 30 NP women were included in this study. All the enrolled RSA patients had experienced at least three consecutive spontaneous early miscarriages ranging from 6 to 10 weeks of gestation. In these patients, we excluded individuals with infections, endocrine or metabolic disorders, anatomic abnormalities, autoimmune diseases, and paternal or maternal chromosomal abnormalities. Detailed information about the study participants was summarized in Table 1.

### Specimen collection

Venous blood samples from RSA patients were taken after a miscarriage, and the blood samples from NP subjects were collected before elective termination of early pregnancy into ethylene diamine tetraacetic acid (EDTA) blood collection tubes (Becton-Dickinson). Samples were processed within 4 hours after collection. PBMCs were isolated by Ficoll density-gradient centrifugation. Plasma was snap frozen and stored at -80°C until assay performance in series. After being extracted through the cervix during dilation and aspiration, decidual tissues were separated immediately and were snap-frozen in liquid nitrogen for quantitative real-time PCR (qRT-PCR) or fixed in 4% paraformaldehyde and embedded in paraffin for immunohistochemical (IHC) and fluorescence *in situ* hybridization (FISH) analyses.

### Cell culture and treatment

293T cells and RAW264.7 cells of the mouse macrophage cell line were obtained from the Cell Bank of Chinese Academy of Sciences. Peritoneal macrophage (PM) was derived from peritoneal dropsy of female C57BL/6 mice (8-10 weeks old) according to previous study 30. In brief, mice were injected with 2 ml 3% fluid thioglycolate medium brewer modified (BD, Franklin Lakes, NJ, USA) to elicit peritoneal macrophage. After 4 days, the mice were sacrificed, and the peritoneal cavity of mice were injected with 5ml pre-chilled 1×PBS buffer followed by massage for 5min. Peritoneal macrophage were immediately collected. All cells were cultured in DMEM (Bioind, Kibbuiz, Israel) containing 10% FBS (Bioind, Kibbuiz, Israel) and 1% penicillin/ streptomycin. The cells were maintained in a humidified atmosphere with 5% CO_2_ at 37°C.

To generate M1 macrophages, RAW264.7 cells and PM were stimulated with 100ng/ml lipopolysaccharides (LPS) (Sigma-Aldrich, St. Louis, MO, USA) and 20ng/ml interferon-γ (IFNγ) (Peprotech, Chicago, IL, USA) for 24h.

### Microarray analysis

RAW264.7 (n= 3) and RAW264.7-derived M1 macrophages induced with 20 ng/mL IFNγ and 100 ng/mL LPS for 24h (n= 3) were used to evaluate miRNAs expression. Total RNA was extracted using TRIzol (Invitrogen, Waltham, MA, USA). The genome-wide analysis of miRNA expression was performed using mouse miRNA, release 21.0 (8*60K, Design ID: 070155). The microarray work was executed by OE Biotech. Co., Ltd (Shanghai, China). The sample labeling, microarray hybridization and washing were performed based on the manufacturer's standard protocols. In brief, total RNA was dephosphorylated, denatured, and then labeled with Cyanine-3-CTP. After purification, the labeled RNAs were hybridized onto the microarray. After washing, the arrays were scanned with the Agilent Scanner G2505C (Agilent Technologies). Feature extraction software (Agilent Technologies, version 10.7.1.1) was used to acquire array images to obtain raw data. Figure signals were transformed to digital signals using Genespring software (Agilent Technologies, version 14.8), and data summarization, normalization and quality control were performed using free miRNA QC Tool software. To determine the significant differentially expressed genes, significance analysis of *t*-test was used. To select the differentially expressed genes, we used threshold values >2 and <-2-fold change and a *P* value <0.05. Besides, a multiple comparison analysis was performed in our microarray data to control false positive rate (FDR), and FDR < 0.05 was considered significant.

### Cell transfection

RAW264.7 and PM cells were maintained in DMEM (Bioind, Kibbuiz, Israel) supplemented with 10% FBS and 1% penicillin/ streptomycin. Cells were cultured in a humidified incubator at 37°C and 5% CO_2_. To investigate the regulatory effect of miR-103 on STAT1, chemosynthetic miR-103 mimics/mimics negative control (NC), miR-103 inhibitor/inhibitor NC (INC) (GenePharma, Shanghai, China) were transfected into cells at a final oligonucleotide concentration of 100 nM with Lipofectamine 2000 (Invitrogen, Waltham, MA, USA). The oligodeoxynucleotide sequences used in this study were shown in Table S1. Instantly, the cells were incubated at 37°C in a 5% CO_2_ atmosphere for 24h. Then, the cells were stimulated by IFNγ (20ng/ml) and LPS (100ng/ml) for 24h to generate M1-polarized macrophage.

### Flow cytometric analysis

Various fluorescent conjugated antibodies to major histocompatibility complex II (MHCII, 12-5321-82), cluster of differentiation 86 (CD86, 17-0862-81), mouse EGF-like module-containing mucin-like hormone receptor-like 1 (F4/80, 17-4801-82) and C-C motif chemokine receptor 7 (CCR7, 12-1971-82) were from eBioscience (Ben Lomond, CA, USA), cluster of differentiation 80 (CD80, 553769) was from BD Biosciences (Franklin Lakes, NJ, USA). RAW264.7 and PM cells were resuspended in 1×PBS and stained with the following conjugated antibodies: CD80, MHCII (IA/IE), CCR7, CD86, F4/80 or their corresponding isotypic control for 30 min at 4°C in the dark. Then, flow cytometry was performed using cell quest software flow cytometer (BD Biosciences, Franklin Lakes, NJ, USA).

Various fluorescent conjugated antibodies to human cluster of differentiation 14 (CD14, 11-0149-42) was from eBioscience (Ben Lomond, CA, USA), CD86 (560957), CD206 (555945) were from BD Biosciences (Franklin Lakes, NJ, USA), tumor necrosis factor-α (TNF-α, 502909) was from Biolegend (San Diego, CA, USA). Decidual tissue from human and mice were cut into 1 mm^3^ pieces, and then flushed repeatedly with 1× PBS using a wide-bore 25-ml pipette to mechanically release cells. The cells were collected and then remove red blood cells with erythrocyte lysate followed by staining with CD14, F4/80, CD86, CD206, MHCII and CD80. For intracellular cytokines TNF-α, cells were stimulated with Cell Stimulation Cocktail Plus Protein Transport Inhibitor (eBiosciences, Ben Lomond, CA, USA) for 6 h at 37°C in 5% CO2, and then fixed and permeabilized with a Fixation/Permeabilization kit (eBiosciences, Ben Lomond, CA, USA). Subsequently, intracellular staining was performed with TNF-α and detected by flow cytometry as above. All data were analyzed by using FlowJo 7.6 (Treestar, USA).

### Immunohistochemical (IHC) staining

For IHC analysis, decidual tissue sections were deparaffinized and endogenous peroxidase activity was blocked with 3% H_2_O_2_ for 30 min in the dark. Antigen retrieval was conducted in 1× citrate buffer in a pressure cooker. STAT1 (14995, Cell Signaling Technology, Danvers, MA, USA) was used as primary antibodies, incubated overnight at 4°C followed by incubation with a biotinylated sheep anti-rabbit secondary antibody. DAB detection kit (ZLI-9018, ZSGB-BIO, Beijing, China) was used for color development and counterstained with hematoxylin. All histological images were acquired using an optical microscope (E100, Nikon, Japan) and analyzed by Image-Toup View software.

### RNA isolation and quantitative real-time PCR (qRT-PCR)

Total RNA was extracted using TRIzol Reagent (Invitrogen, Waltham, MA, USA) following the manufacturer's instruction. RNA was reverse transcribed with the miRNA 1st Strand cDNA Synthesis Kit (Vazyme, Nanjing, China) for miRNA or the PrimeScript RT reagent Kit (Toyobo, Osaka, Japan) for mRNA per the manufacturer's instructions. qRT-PCR using SYBR Green (Invitrogen, Waltham, MA, USA) was performed on Applied Biosystems 7500 instrument (Applied Biosystems, Foster, USA). For miRNA and mRNA analysis, the primer sequences were shown in Table S2. For each sample, the amplification reaction was performed in triplicate. Relative RNA quantification was performed via the comparative 2^-ΔΔCt^ method. The relative expression levels of miRNA were normalized to that of internal control U6, while the relative expression of genes was normalized to the level of *ACTB* expression in each sample.

### Western blot analysis

Cells and decidual tissues were lysed in RIPA buffer (Thermo Scientific, Massachusetts, USA) with protease and phosphatase inhibitor (Thermo Scientific, Massachusetts, USA) and analyzed by western blot as described previously 31. Protein concentration was determined by BCA protein assays kit (Thermo Scientific, Massachusetts, USA) according to the manufacturer's instruction. Antibodies against STAT1 (14995, Cell Signaling Technology, Danvers, MA, USA), phosphorylated STAT1 (p-STAT1, 9167, Cell Signaling Technology, Danvers, MA, USA), inducible nitric oxide synthase (iNOS, 2982, Cell Signaling Technology, Danvers, MA, USA), IRF1 (8478, Cell Signaling Technology, Danvers, MA, USA), interferon regulatory factor-5 (IRF5, 20261, Cell Signaling Technology, Danvers, MA, USA), interferon regulatory factor-8 (IRF8, 5628, Cell Signaling Technology, Danvers, MA, USA) and GAPDH (ab181603, Abcam, Cambridge, MA, USA) were used according to the manufacturer's instruction.

### Dual luciferase reporter assay

The TargetScan (http://www.targetscan.org/vert_71/) predictions indicated that there were complementary binding sites between *STAT1* mRNA 3' UTR and miR-103, and luciferase reporter assay was performed to identify the combination. The wild-type (WT) and mutant (MUT) human *STAT1* mRNA 3'UTR luciferase reporter vectors were constructed by amplifying human WT or MUT *STAT1* mRNA 3'UTR and cloning into pGL3-3M-Luc vector (Promega, Madison, WI, USA), respectively. 293T cells were co-transfected with WT or MUT luciferase reporter plasmid and the miR-103 mimics or NC with final concentration of 100nM. After 24h, the cells were collected for application in the Dual-Luciferase® Reporter Assay System (Promega, Madison, WI, USA) using a GloMax 20/20 Luminometer (Promega, Madison, WI, USA) under recommended condition. Ratios of firefly luciferase luminescence relative to renilla luciferase luminescence were calculated.

### Immunofluorescence (IF)

RAW264.7 cells were plated in 24-well culture plates at a concentration of 5×10^4^ cells/well and transfected with miR-103 mimics or NC. After 24 h, the cells were stimulated by IFNγ and LPS for 24h. Thereafter, the treated cells were fixed with 4% paraformaldehyde at room temperature for 30 minutes and then incubated in 10% normal mice serum for 30 minutes. The cells were then incubated with STAT1 antibody (Cell Signaling Technology, Danvers, MA, USA) or IRF1 (8478, Cell Signaling Technology, Danvers, MA, USA) overnight at 4°C. The secondary antibodies FITC Mouse Anti-Rabbit IgG (BOSTER, Wuhan, China) was used for 1 hour. Sections were counterstained with DAPI and analyzed with a fluorescence microscope (IX73, Olympus, Japan).

### Animal experiments

Eight-week-old CBA/J female, DBA/2 male and BALB/c male mice were obtained from Beijing Biotechnology Co., Ltd and kept in a specific pathogen-free animal facility. CBA/J females were mated to BALB/c males to establish a NP model (CBA/J× BALB/c) and to DBA/2 males to establish an RSA (CBA/J× DBA/2) model as described previously 9. The animal experiments were carried out in accordance with the guidelines for the Care and Utilization of Laboratory Animals (Institute of Basic Medicine, Shandong Provincial Hospital Affiliated to Shandong First Medical University) and were approved by the Institutional Animal Care and Use Committee of the Institute of Basic Medicine, Shandong Provincial Hospital Affiliated to Shandong First Medical University.

Mice were inspected every morning for vaginal plugs. The day when a plug became visible was designated as day 0.5 of pregnancy. Normal pregnant mice were injected with agomir NC (Ribobio, Guangzhou, China). RSA mice were randomly divided into two groups: (1) RSA agomir NC group, mice were injected with agomir NC; (2) RSA agomiR-103 group, mice were injected with agomiR-103 (Ribobio, Guangzhou, China). All mice were injected via tail vein every 3 days at the dose of 10 nmol per mouse in 200 μl of saline respectively from day 0.5 until sacrificed according to previous study 21. Mice were sacrificed on day 11.5, and the embryo resorption rate was calculated. Decidual tissues were collected for western blot, qRT-PCR and flow cytometry analysis respectively. Unpregnant mice were excluded and ten mice from each group were selected for further analysis.

### Fluorescence *in Situ* Hybridization (FISH)

FISH assay was executed to observe the location and expression of miR-103 in the decidual tissues of RSA patients and NP subjects. The decidual tissues were fixed with 4% paraformaldehyde at room temperature and made into paraffin slices at 5μm thickness. The paraffin slices were hybridized with specific Cy3-labeled miR-103 probes (Cy3-5'-TCATAGCCCTGTACAATGCTGCT-3') (GenePharma, Shanghai, China) at 37°C overnight. 4,6-diamidino-2-phenylindole (DAPI) was used for cell nucleus counterstain and the images were acquired with laser scanning confocal microscope (FV3000, Olympus, Japan). All procedures were conducted according to the manufacturer's protocol (Genepharma, Shanghai, China).

### Fetal resorption rate analysis

Because of ischemia, hemorrhage and necrosis, the volume of resorbed embryos was significantly smaller than those of normal embryos. In addition, the color of embryo is different between the resorbed and normal embryos: the reabsorbed embryo is deep brown and the normal embryo is pink. The number of re-absorbed embryos and viable embryos were calculated by embryo absorptivity (R) = Re/(Re+ F) as previously described 32, where Re is the number of re-absorbed embryos, and F is the number of viable embryos.

### Statistical analysis

Unless otherwise stated, all experiments were performed at least three independent times. Values are presented as the mean± SEM. The 2-tailed Student *t* test was used to compare the data between any two groups through the normality and equal variance tests. If data for either normality or variance tests failed, nonparametric Mann-Whitney U test was used. For multiple comparisons, one-way ANOVA on ranks with Bonferroni post hoc test were used. If data did not pass either test, then nonparametric Kruskal Wallis test with Dunn post hoc test was used. Data was plotted using GraphPad Prism 6.0 software (GraphPad Software, Inc., CA, USA). Statistical analyses were carried out with SPSS software (version 16.0). Correlations were analyzed by Pearson correlation. The diagnostic value was evaluated using the receiver operating characteristic (ROC) curve. *P* <0.05 was considered significant.

## Results

### M1 macrophage is increased in decidua of RSA patients and positively correlated with STAT1

**Figure 1:**
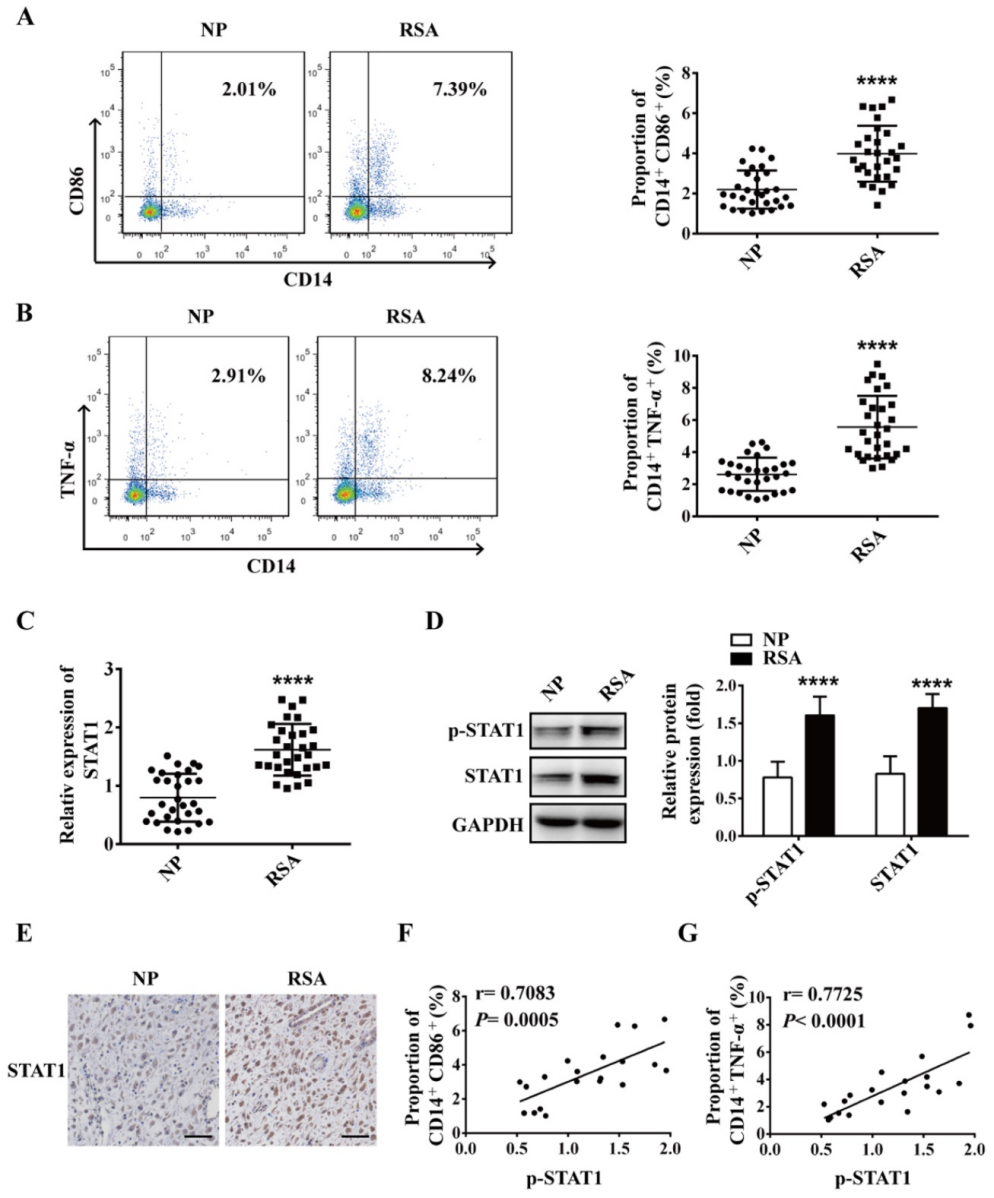
** M1 macrophage and STAT1 were excessive in RSA patients.** (**A-B**) The dot plot represents labeling of CD14^+^ CD86^+^ and CD14^+^ TNFα^+^ (M1) cells by flow cytometry in decidua of NP subjects (n= 30) and RSA patients (n= 30). (**C**) qRT-PCR analysis of *STAT1* expression in the decidua of NP subjects (n= 30) and RSA patients (n= 30). (**D**) STAT1 and p-STAT1 protein levels were measured in decidua of NP subjects (n= 10) and RSA patients (n= 10) by western blot. (**E**) Representative IHC staining images of STAT1 in the decidua of NP and RSA patients (Scale bar, 50 µm, 200×). (**F**) Correlation between p-STAT1 and the proportion of CD14^+^ CD86^+^ in decidua of NP subjects (n= 10) and RSA patients (n= 10). (**G**) Correlation between p-STAT1 and the proportion of CD14^+^ TNF-α^+^ in decidua of NP subjects (n= 10) and RSA patients (n= 10). Values were listed as the mean± SEM. **** P< 0.0001.

To explore the role of macrophage in RSA, macrophages in decidua of both RSA patients and NP group were detected by flow cytometry. The data showed that compared with NP group, the proportion of M1 macrophages (CD14^+^CD86^+^ and CD14^+^TNF-α^+^) increased significantly in the decidua of RSA patients (Figure 1A-B), however, the proportion of M2 macrophages (CD14^+^CD206^+^) decreased significantly in the decidua of RSA patients (Figure S1). Meanwhile, the mRNA level of STAT1 and protein levels of STAT1 and p-STAT1 up-regulated markedly in the decidua of RSA patients compared with NP group (Figure 1C-D). Similar results were noted in the IHC for STAT1 protein level in the decidua of RSA patients (Figure 1E). Interestingly, the proportion of CD14^+^CD86^+^ and CD14^+^TNF-α^+^ cells (M1 macrophages) were positively-related to p-STAT1 expression (Figure 1F-G). These results suggest that elevated M1 macrophages may be mediated by up-regulated STAT1 in RSA.

### miR-103 was down-regulated in RSA patients and negatively correlated with STAT1 expression

**Figure 2:**
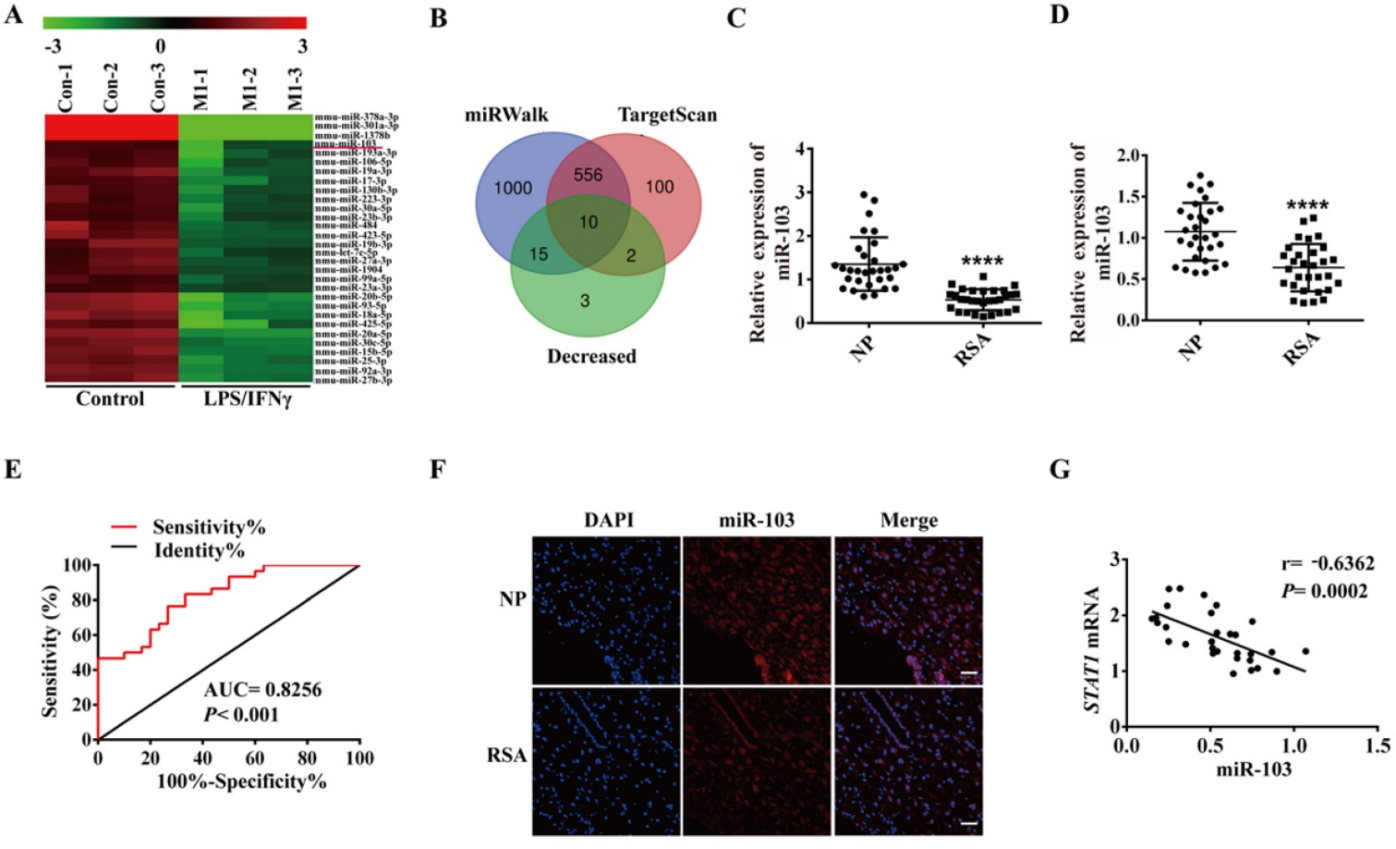
** miR-103 was down-regulated in RSA patients and negatively correlated with STAT1 expression.** (**A**) Decreased expression of 30 miRNAs (*P* <0.05 and fold change >2) detected in the RAW264.7 and RAW264.7- derived M1 macrophage through miRNA microarray analysis. (**B**) Schematic representation showing the 10 predicted potential miRNAs that might target STAT1 screened by using decreased miRNA in microarray assay of RAW264.7 derived M1 macrophage, TargetScan and miRWalk prediction. (**C**) The expression level of miR-103 in decidua tissues of NP (n= 30) and RSA case (n= 30). (**D**) The expression level of miR-103 in PBMC of NP (n= 30) and RSA case (n= 30). (**E**) Diagnostic value of miR-103 in PBMC for RSA was assessed by ROC curve (n= 30). (**F**) FISH was performed to observe the location and expression level of miR-103 in decidual tissues (Scale bar, 50 µm, 200×). (G) Correlation between *STAT1* mRNA and miR-103 in 30 decidua tissues of RSA. Values were listed as the mean± SEM. **** P< 0.0001.

In order to elucidate the role of miRNAs in M1 polarization, RAW264.7 was stimulated with LPS and IFNγ to generate M1 macrophages and global miRNA expression profiles were detected by using miRNA microarray assay. A total of 51 dysregulated miRNAs were detected by at least 2-fold and *P* < 0.05 in the LPS plus IFNγ induced RAW264.7, including 21 up-regulated and 30 down-regulated miRNAs (Table S3). The down-regulated miRNAs were showed as heatmap (Figure 2A). We used TargetScan, miRWalk database and Chip to get the list of potential miRNAs that might target STAT1. Three lists of miRNAs were overlapped, and 10 miRNAs (miR-103, miR-23b-3p, miR-20b-5p, miR-93-5p, miR-106b-5p, miR-20a-5p, miR-17-3p, miR-23a-3p, miR-18a-5p and miR-378b) were revealed to likely target STAT1 (Figure 2B). Subsequently, qRT-PCR was performed to verify the 10 down-regulated miRNAs in the human decidual samples (Figure 2C and Figure S2A-I). Among 10 down-regulated miRNAs, miR-103 was the most downregulated in the human decidual samples and selected for further analysis. To control false positive rate, a multiple comparison analysis was performed in our microarray data, and the FDR value of miR-103 was 0.01810276. Meanwhile, the expression of miR-103 was also identified in human PBMC samples and found that miR-103 expression was robustly decreased in RSA patients compared with that of NP group (Figure 2D). ROC analysis revealed that miR-103 could sensitively discriminate RSA in PBMCs with an area under the curve (AUC) of 0.8256 (95% confidence interval [CI]: 0.7244- 0.9267) (Figure 2E). FISH assay identified that miR-103 (red) expression decreased obviously in the decidua of RSA patients (Figure 2F). Furthermore, Pearson correlation analysis showed that there was a significantly negative correlation between miR-103 and STAT1 (Figure 2G). Collectively, these findings indicated that downregulated miR-103 might promote M1 macrophages differentiation by disinhibiting STAT1 and could serve as a diagnostic marker for RSA.

### miR-103 inhibits M1 macrophage polarization by suppressing STAT1/IRF1 signal pathway *in vitro*

**Figure 3:**
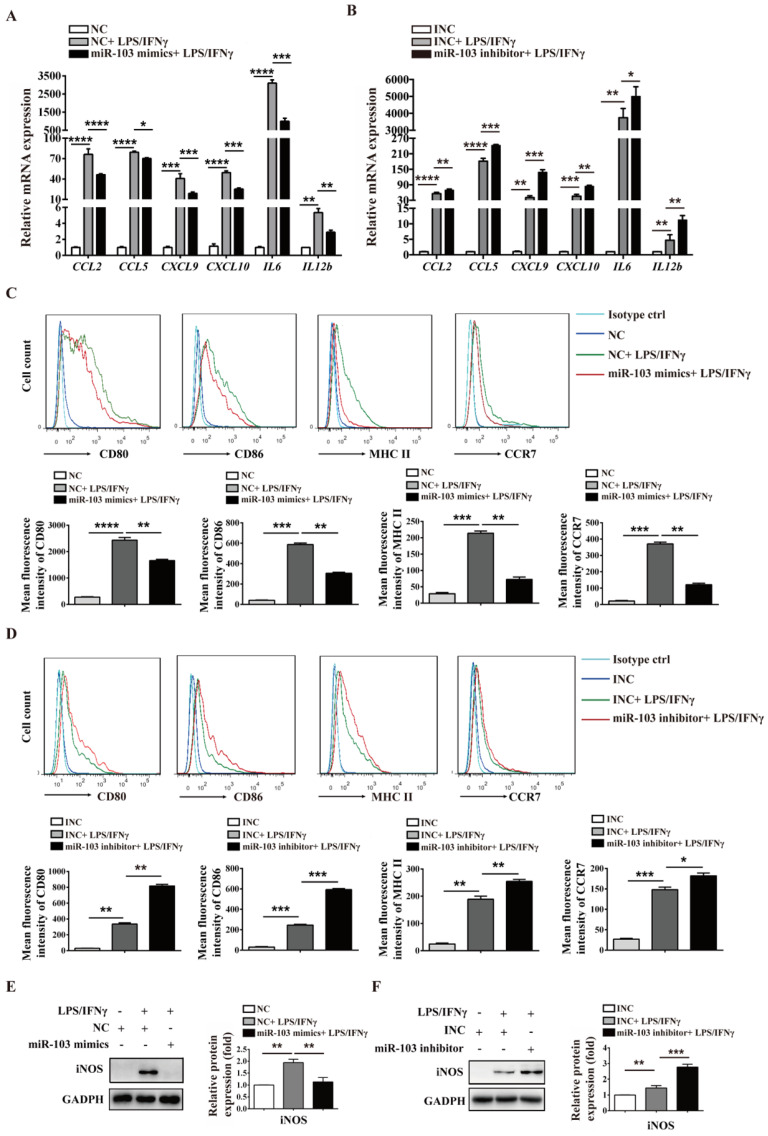
** miR-103 reduces M1-polarized RAW264.7 macrophages.** RAW264.7 cells were transfected with miR-103 mimics/NC or miR-103 inhibitor/INC for 24h, and then stimulated with or without LPS/IFNγ for 24 h. (**A-B**) The mRNA expression of M1 markers *CCL2*, *CCL5*, *CXCL9*, *CXCL10*,* IL6*, *IL12b* were determined using qRT-PCR. (**C-D**) The surface levels of CD80, CD86, MHCII, and CCR7 were analyzed by flow cytometry. (**E-F**) Western blot analysed the protein levels of iNOS. Values were listed as the mean± SEM. *P< 0.05, **P< 0.01, ***P< 0.001, ****P< 0.0001.

**Figure 4:**
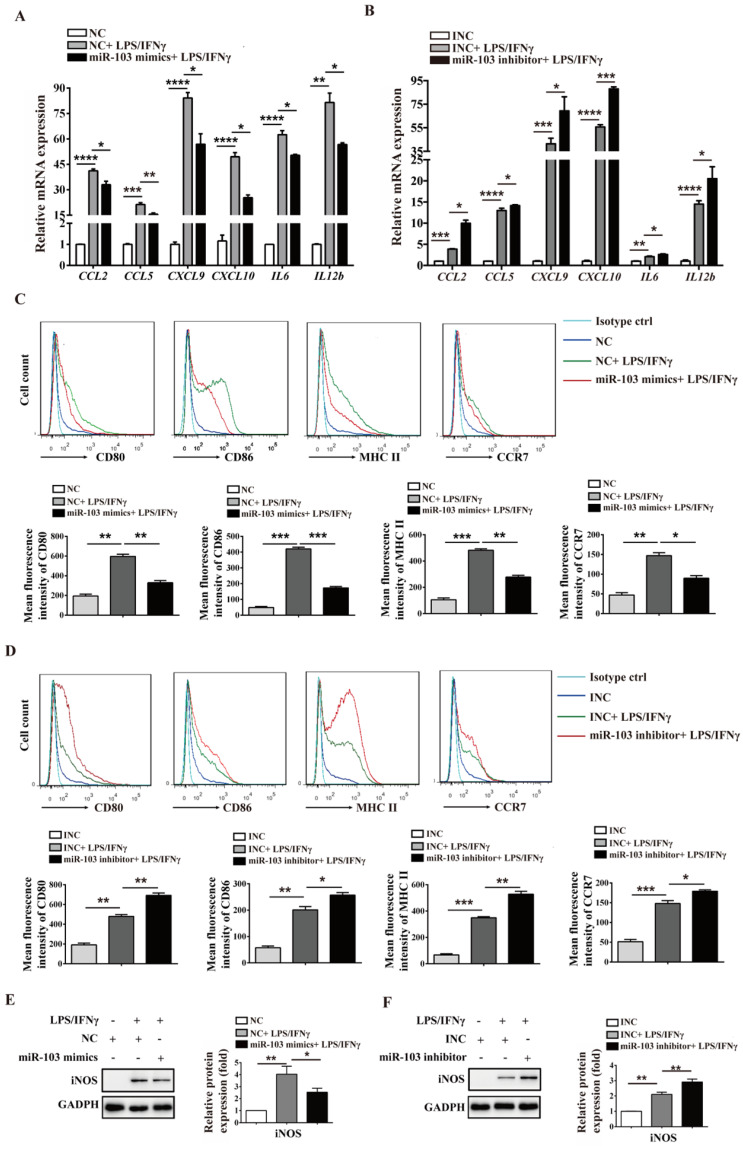
** miR-103 reduces M1-polarized PM macrophages.** PM cells were transfected with miR-103 mimics/NC or miR-103 inhibitor/INC for 24h, and then stimulated with or without LPS/IFNγ for 24 h. (**A-B**) The mRNA expression of M1 markers* CCL2*, *CCL5*, *CXCL9*, *CXCL10*, *IL6*, *IL12b* were determined using qRT-PCR. (**C-D**) Surface expression of CD80, CD86, MHCII, and CCR7 were analyzed by flow cytometry, (**E-F**) Western blot analysed the protein levels of iNOS. Values were listed as the mean± SEM. *P< 0.05, **P< 0.01, ***P< 0.001, ****P< 0.0001.

To investigate the potential biological effect of miR-103 on the polarization of M1 macrophages, we altered miR-103 expression and determined the expression of M1 markers in both RAW264.7- and PM-derived M1 macrophages *in vitro*. The data showed that miR-103 mimics enhanced while the inhibitor suppressed miR-103 expression effectively both in RAW 264.7 and PM cells (Figure 3SA-D). Moreover, compared with NC group, treatment with LPS and IFNγ significantly increased M1 markers such as chemokine (C-C motif) ligand 2 (*CCL2*), chemokine (C-C motif) ligand 5 (*CCL5*), chemokine (C-X-C motif) ligand 9 (*CXCL9*), chemokine (C-X-C motif) ligand 10 (*CXCL10*), interleukin 6 (*IL6*) and interleukin 12b (*IL12b*) expression, however, miR-103 overexpression inhibited the M1 macrophage-inducing effects of LPS and IFNγ markedly (Figure 3A and Figure 4A). In contrast, miR-103 knockdown could enhance the M1 polarization effectively induced by LPS and IFNγ (Figure 3B and Figure 4B). Meanwhile, in comparison with NC group, the mean fluorescence intensity (MFI) of CD80, CD86, MHCII, CCR7 (Figure 3C and Figure 4C) and protein expression of iNOS (Figure 3E and Figure 4E) were also found to be reduced significantly in miR-103 overexpression group, while, down-regulation miR-103 showed the opposite effects (Figure 3D, 3F and Figure 4D, 4F). Taken together, these data suggested that miR-103 can suppresses M1 macrophage polarization and encourage us to further explore the underlying mechanism.

**Figure 5:**
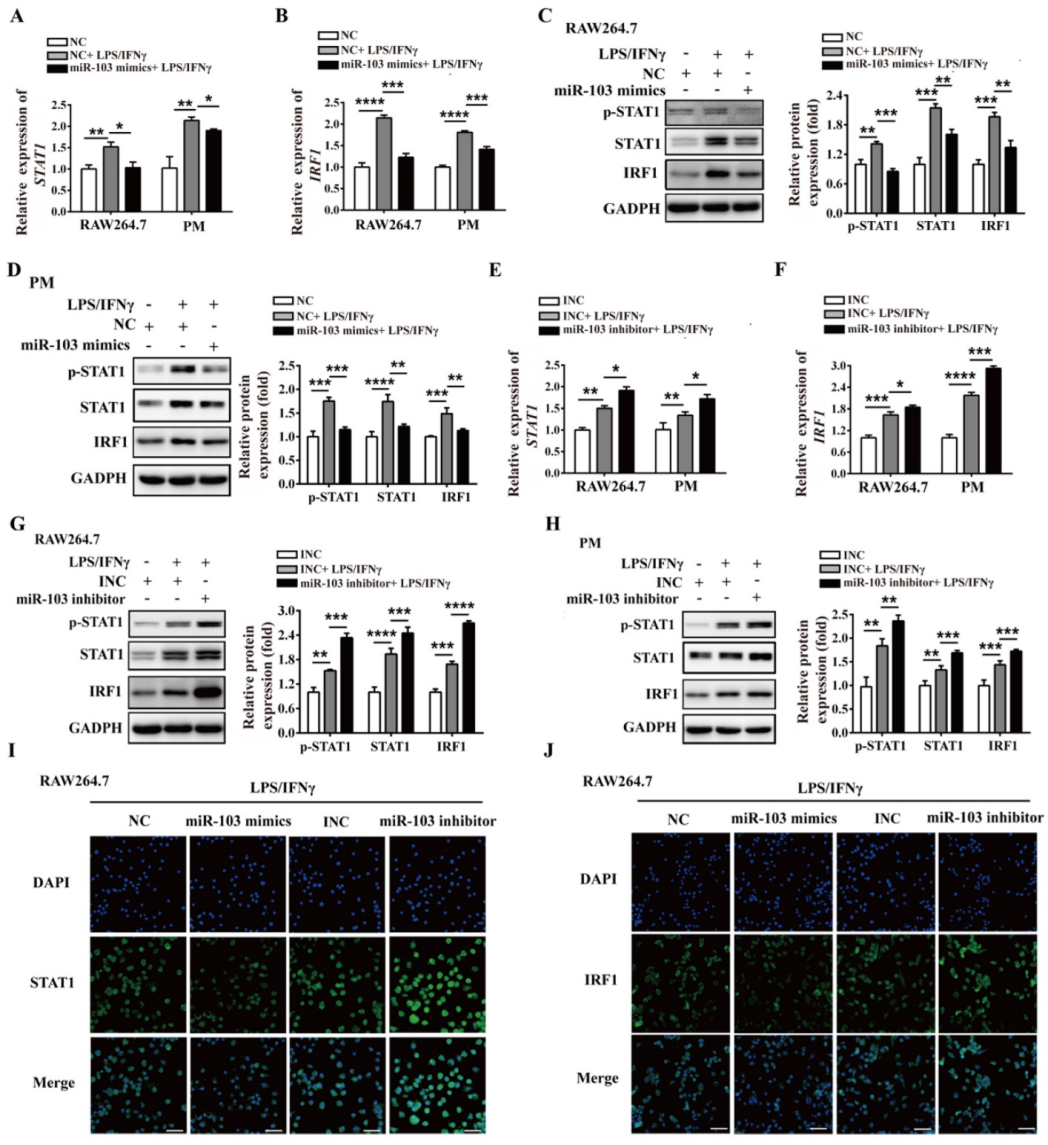
** miR-103 inhibits the STAT1/IRF1 signal pathway.** RAW264.7 and PM cells were transfected with miR-103 mimics/NC or miR-103 inhibitor/INC, after 24h, the cells were stimulated with or without LPS/IFNγ for 24 h. (**A-B**) *STAT1* and *IRF1* mRNA expression were detected in RAW264.7 and PM cells transfected with miR-103 mimics or NC by qRT-PCR. (**C-D**) The protein levels of STAT1, p-STAT1 and IRF1 were measured in RAW264.7 and PM cells transfected with miR-103 mimics or NC by western blot. (**E-F**) *STAT1* and *IRF1* mRNA expression were detected in RAW264.7 and PM cells transfected with miR-103 inhibitor or INC by qRT-PCR. (**G-H**) STAT1, p-STAT1 and IRF1 protein levels were measured in RAW264.7 and PM cells transfected with miR-103 inhibitor or INC by western blot. (**I-J**) Expression of STAT1 and IRF1 were detected in RAW264.7 cells transfected with miR-103 mimics/NC or miR-103 inhibitor/INC by immunofluorescence. DAPI was used to stain the cell nucleus (Scale bar, 50 µm, 200×). Values were listed as the mean± SEM. *P< 0.05, **P< 0.01, ***P< 0.001, ****P< 0.0001.

Previous studies demonstrated that STAT1 is a key effector in M1 polarization, and STAT1 activation by IFNγ in macrophages results in their polarization toward M1 phenotype 33. In order to study whether miR-103 affects M1 macrophage activation through STAT1 pathway, we altered miR-103 expression and determined the levels of STAT1 and STAT1 downstream genes *IRF1*, *IRF5*, *IRF8* in both RAW264.7- and PM-derived M1 macrophages. Interestingly, we found that overexpression of miR-103 reduced *STAT1*, *IRF1* and *IRF5* mRNA expression efficiently without affecting that of *IRF8* (Figure 5A-B and Figure S4A). Meanwhile, miR-103 mimics transfection and inhibited the protein level of STAT1, p-STAT1, and IRF1 without affecting that of IRF5, IRF8 (Figure 5C-D and Figure S4B). In comparison, down-regulated miR-103 enhanced STAT1 phosphorylation and increased mRNA and protein level of STAT1 and IRF1 without affecting that of IRF5, IRF8 (Figure 5E-H and Figure S4C-D). In addition, IF revealed that overexpression of miR-103 markedly decreased the STAT1 and IRF1 expression in RAW264.7 cells treated with LPS/IFNγ, and vice versa (Figure 5I-J). In summary, these data suggested that miR-103 may suppress M1 macrophage polarization by inhibiting STAT1/IRF1 signaling pathway.

### *STAT1* is a direct target gene of miR-103

**Figure 6:**
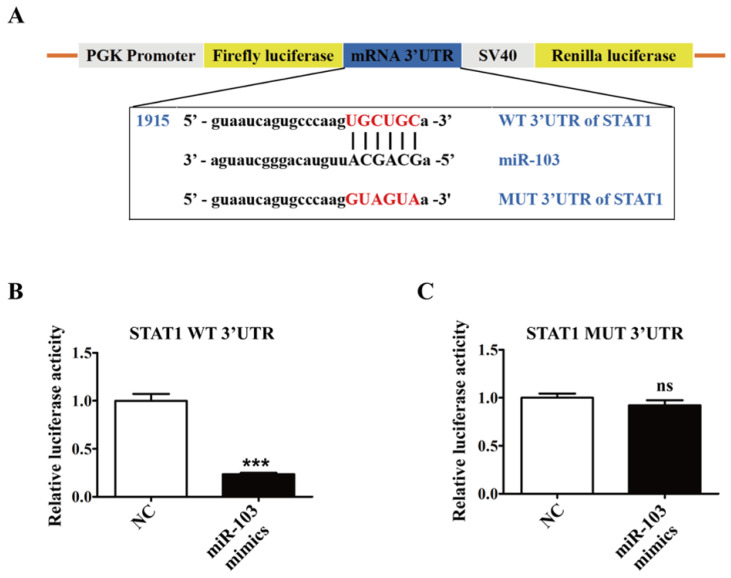
***STAT1* is a direct target of miR-103.** (**A**) Schematic representation of miR-103 putative binding sequence in the 3'UTR of *STAT1*, luciferase activities of wild-type (WT) and mutant (MUT) constructs. (**B-C**) The luciferase activity was determined by co-transfecting the vectors (*STAT1* 3'UTR-WT or *STAT1* 3'UTR-MUT) combined with NC, miR-103 mimics into 293T cells. Values were listed as the mean± SEM. ***P<0.001; ns means no statistical difference.

To validate the post-transcriptional suppressive effect of miR-103 on *STAT1*, WT and MUT 3'UTR for *STAT1* were cloned into a pGL3-3M-Luc vector and co-transfected with miR-103 mimics or NC for dual-luciferase reporter assay (Figure 6A). The results showed that miR-103 mimics markedly reduced luciferase reporter activity of WT 3'UTR plasmid for* STAT1*, without affecting that of MUT 3'UTR plasmid (Figure 6B-C). Therefore, the data proved that *STAT1* was the direct target gene of miR-103 and down-regulated miR-103 contributed to elevated STAT1 in RSA.

### Overexpression of STAT1 reverses the inhibitory effect of miR-103 on M1 polarization *in vitro*

**Figure 7:**
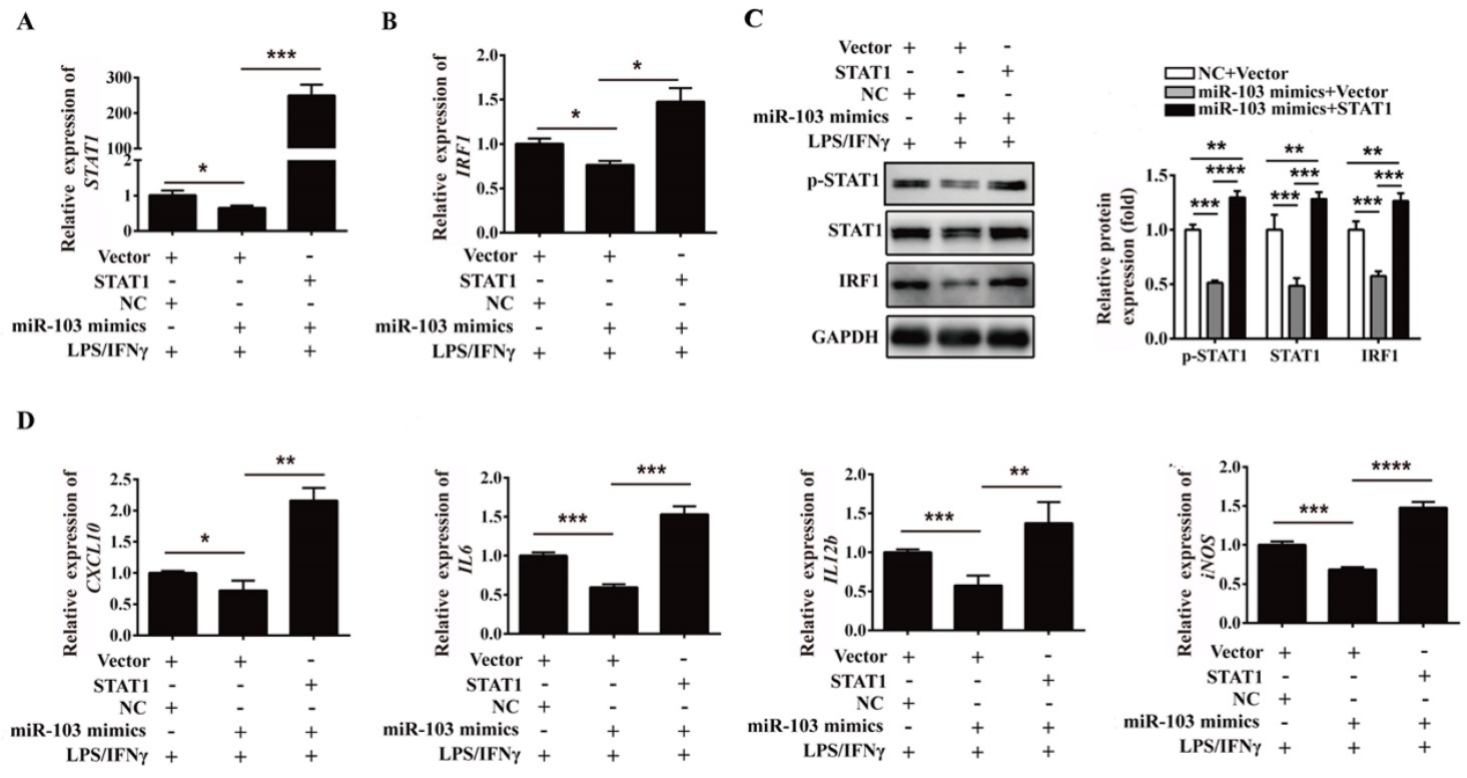
** Overexpression of STAT1 can reverse the inhibitory effect of miR-103 on M1 polarization.** RAW264.7 cells were co-transfected with miR-103 mimics, STAT1 plasmid or NC, vector for 24 h, followed by treating with LPS plus IFNγ for 24 h. (**A-B**) *STAT1* and *IRF1* mRNA levels were assessed by qRT-PCR. (**C**) p-STAT1, STAT1 and IRF1 protein levels were assessed by western blot. (**D**) mRNA expression level of M1 macrophages makers *CXCL10*, *IL6*, *IL12b*, *iNOS* were detected by qRT-PCR. Values were listed as the mean± SEM. *P < 0.05, **P < 0.01, ***P < 0.001, ****P < 0.0001.

In order to further address whether miR-103 suppresses M1 macrophage polarization by inhibiting STAT1/IRF1 signaling pathway, rescue analysis was executed. RAW264.7 cells were co-transfected with miR-103 mimics and *STAT1* overexpressed plasmid and then treated with LPS/IFNγ. Consistent with the above results, overexpressed miR-103 could reduce STAT1 phosphorylation and inhibited mRNA and protein level of STAT1, IRF1 efficiently, but this effect could be effectively reversed by overexpressed *STAT1* (Figure 7A-C). Moreover, mRNA expression of *CXCL10*, *IL6*,* IL12b* and* iNOS* could also be reversed by overexpressed *STAT1* (Figure 7D). Collectively, these findings suggested that miR-103 inhibited M1 polarization by suppressing STAT1 and the disinhibition of STAT1/IRF1 signaling pathway induced by downregulated miR-103 may be the underlying mechanism of increased M1 polarization in RSA.

### Upregulated miR-103 decreases embryo resorption by suppressing M1 differentiation *in vivo*

**Figure 8:**
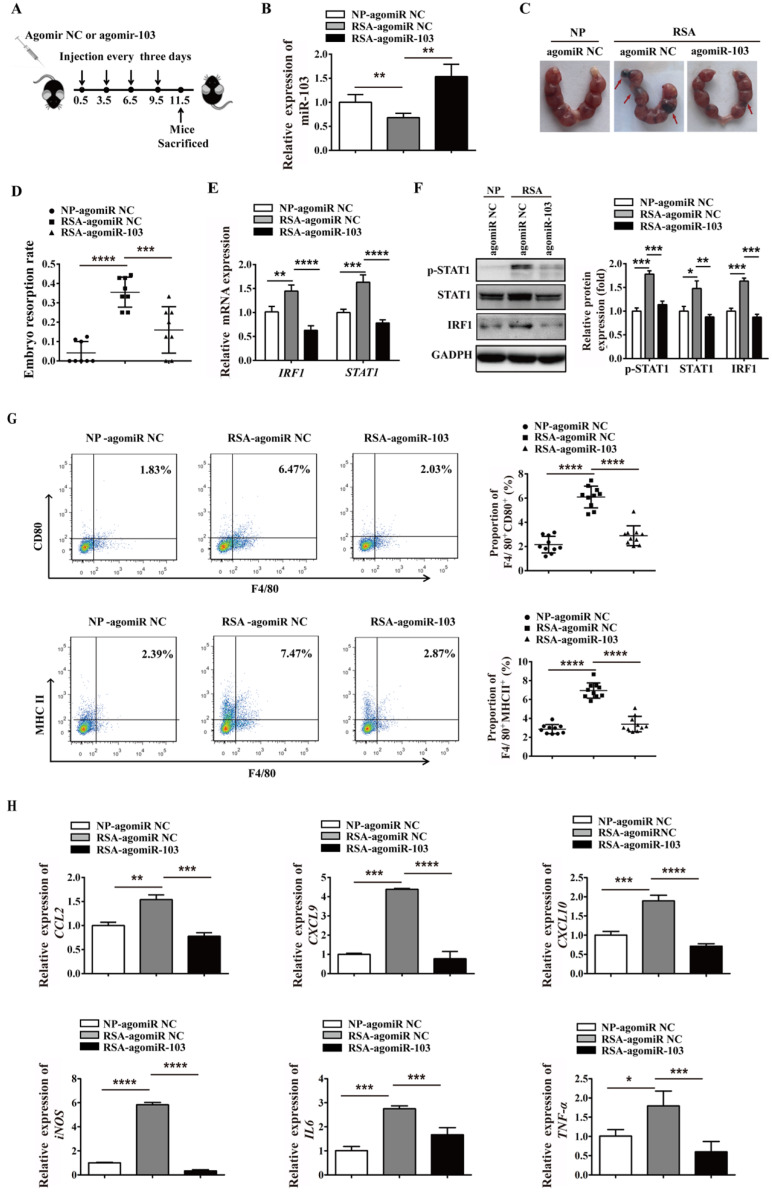
** miR-103 suppresses embryo resorption rate and M1 macrophages polarization *in vivo*.** Mice were inspected every morning for vaginal plugs. The day when a plug became visible was designated as Day 0.5 of pregnancy. NP mice and RSA mice were administrated 10 nmol agomiR NC or agomiR-103 on Day 0.5, 3.5, 6.5, 9.5 via tail vein, and execute mice on Day 11.5 of pregnancy. (**A**) Treatment regime of agomiR-103 or agomiR NC and timeline for the measurement of parameters. (**B**) Relative expression of miR-103 was measured by qRT-PCR in the decidua of pregnant mice (n= 10). (**C-D**) Embryo resorption rate of three group mice, arrows indicate the embryo resorption (n= 10). (**E-F**) The mRNA level of STAT1, IRF1 and protein level of p-STAT1, STAT1 and IRF1 were detected in the decidua of pregnant mice (n= 10). (**G**) Dot plot represents labeling of F4/80^+^CD80^+^ (M1) and F4/80^+^MHCII^+^ (M1) cell by flow cytometry in the decidua of pregnant mice (n= 10). (**H**) Relative expression of *CCL2*, *CXCL9*, *CXCL10*,* iNOS*, *IL6*, *TNF-α* was analysed by qRT-PCR in the decidua of pregnant mice (n= 10). Values were listed as the mean± SEM. *P< 0.05, **P< 0.01, ***P< 0.001, ****P< 0.0001.

To extend the study of the precise function role of miR-103 in RSA, we introduced agomiR-103 to imitate the effect of miR-103 *in vivo*. NP mouse and RSA mouse received agomiR NC and agomiR-103 respectively to construct miR-103 overexpression model (Figure 8A). Consistent with RSA patients, the expression of miR-103 in decidua of RSA mice was decreased compared with NP mice, but after agomiR-103 treated, the expression of miR-103 in decidua increased significantly (Figure 8B). Interestingly, it was found that administration of agomiR-103 could obviously decrease embryo resorption rate in RSA mice compared with RSA mice treated with agomiR NC (Figure 8C-D). Moreover, increased STAT1 phosphorylation, mRNA and protein level of STAT1, IRF1 were found in the decidua of RSA mice treated with agomiR NC compared with agomiR NC-treated NP mice, but after agomiR-103 treated the rising trend was decreased obviously (Figure 8E-F). Furthermore, flow cytometry assay showed that the proportion of F4/80^+^CD80^+^ and F4/80^+^MHCII^+^ M1 macrophages increased significantly in the decidua of RSA mice treated with agomiR NC, while, they were significantly reduced in that of the agomiR-103-treated RSA mice (Figure 8G). Similarly, the expression of M1 makers such as *CCL2*, *CXCL9*, *CXCL10*,* iNOS*, *IL6*, and *TNF-α* increased significantly in the decidua of agomiR NC-treated RSA mice, while, they were decreased effectively after the treatment of agomiR-103 (Figure 8H)*.* Taken together, these data suggested that miR-103 reduced M1 macrophages polarization by inhibiting STAT1/IRF1 pathway and thereby decreasing embryo resorption rate of mice. miR-103/STAT1/IRF1 axis maybe a potential therapeutic target for RSA.

## Discussion

Human pregnancy is considered to be a unique immunological paradigm. On one hand, it requires maternal tolerance to the semi-allogenic fetus; on the other hand, it also needs the maintenance of an appropriate immune status to protect both mother and fetus against pathogens 34. Failure to promote immune tolerance to paternal antigens may result in pregnancy loss 35. Accumulating evidences have demonstrated that macrophages are closely associated with homeostatic and tolerogenic properties and play important roles in the establishment and maintenance of pregnancy 34-36. Moreover, macrophages activation towards a more M1 phenotype has been associated with the RSA 37. Unfortunately, our understanding of the underlying mechanism of macrophages polarization in normal pregnancy and pregnancy-associate diseases is still limited. Consistent with previous studies, we found an increased percentage of M1 macrophages in the decidua of RSA patients. Importantly, we provided the evidence that downregulated miR-103 plays an important role as a promoter in M1 macrophage polarization by disinhibiting STAT1/IRF1 pathway. We identified that miR-103 regulates STAT1 expression by directly interacting with its 3'UTR. Furthermore, we demonstrated that overexpressed miR-103 suppressed M1 macrophage polarization and thereby alleviating embryo resorption rate *in vivo*. Therefore, miR-103 may serve as a promising diagnostic marker and therapeutic target for RSA.

The maternal-fetal interface is heavily infiltrated by immune cells throughout pregnancy. Macrophages account for 20-30% of the total decidual immune cells in the first trimester, indicating the crucial role of macrophages in the establish and maintenance of pregnancy 38,39. Macrophages are broadly categorized into M1 and M2 macrophages based on the production of specific factors, expression of cell surface markers, and biological activities 40. M1-polarized macrophages are more effective at antigen clearance and switching the T-cell response toward T helper-1 (Th1) immune response, while M2 cells have immunosuppressive capacities, contribute to tissue remodeling, and promote T helper-2 (Th2) or antibody-mediated immune responses 41. It has been demonstrated that gene expression profiling of human decidual showed an M2-dominant macrophages phenotype in the first trimester, characterized by CD209, and expression of anti-inflammatory genes 42. Macrophages activation towards a more M1 phenotype has been associated with the pathology of recurrent miscarriage 8,9,37. In line with previous studies, in our research, we found the proportion of M1 macrophages increased, however the proportion of M2 macrophages decreased significantly in the decidua of RSA patients.

miRNAs are small non-coding RNAs that regulate gene expression by binding to the 3'UTR of their target genes in a sequence-specific manner, thereby decreasing the gene expression 43. miRNAs have been demonstrated to play crucial roles in most biological and physiological processes, including cell growth and differentiation, metabolism, immunity, cancer, and autoimmune disorders 44-46. Recently, increasing evidences have suggested that miRNAs are associated with M1 macrophage polarization, such as miR-181a, miR-130a, let-7c, miR-99a 26-29. Studies have demonstrated that miRNAs also play essential roles in RSA such as miR-365, miR-133a, miR-520 and miR-184 19-22. Although a number of reports have proved that miRNAs play essential roles in the occurrence of RSA, the regulatory effect of miRNAs on M1 differentiation in RSA has not been fully investigated. Our study focused on revealing miRNAs potentially functional in M1 macrophage polarization during RSA occurrence and uncovered for the first time that STAT1/IRF1 signaling mediated M1 polarization could be targeted to protect form embryo loss by miR-103 overexpression.

miR-103 is a member of the miR-15/107 family 47, located on human chromosome 5q34 region, and mice chromosome 11A4 region. It has been extensively studied and confirmed to play important roles in various types of cancer such as gastric cancer, colorectal cancer, prostate cancer, and hepatocellular carcinoma 48-51. Besides, miR-103 is up-regulated during adipocyte differentiation, and ectopic expression of miR-103 in preadipocytes accelerated adipogenesis 52,53. More importantly, during adipocyte differentiation miR-103 could increase peroxisome PPARγ activation 52. It is known that PPARγ is a transcriptional factor which positively correlated with the expression of M2 markers such as Mrc1 and IL-10 which could control the inflammatory response by negatively interfering with pro-inflammatory signaling pathways 54. Moreover, studies of coronary atherosclerosis and abdominal aortic aneurysm showed that miR-103 played a novel role in negatively regulating inflammatory pathways through targeting chemokine (C-C motif) ligand 13 (*CCL13*) and ADAM metallopeptidase domain 10 (*ADAM10*) respectively 55,56. Those studies suggested that miR-103 is involved in anti-inflammatory effects and may play important roles in macrophage differentiation. In our study, we found that miR-103 expression was substantially lower in both decidua and PBMCs of RSA patients, and ROC analysis demonstrated that miR-103 could sensitively discriminate RSA patients from NP subjects. Meanwhile, the miRNA profile revealed that miR-103 was downregulated in RAW264.7-derived M1 macrophages. These findings indicated that miR-103 might be used as a diagnostic marker for RSA. Importantly, we found that overexpression of miR-103 suppressed M1 polarization significantly, while miR-103 knockdown displayed an opposite effect.

STAT1 is a member of the STAT family encoded by STAT1 gene located on human chromosome 2q32.2 57. IRF1 is a transcriptional regulator at the downstream of STAT1 and STAT1/ IRF1 signaling pathway played an important role in M1 polarization 13,14. Accumulating evidence showed that STAT1 is a central transcription factor in modulating macrophage polarization and STAT1 activation will skew macrophage function toward the M1 phenotype 10. In human primary macrophages derived from CD14^+^ PBMCs, poly (ADP-ribose) polymerase family member 14 (PARP14) silencing accelerated IFNγ induced STAT1 phosphorylation and improved M1 macrophage activation 58. Ning Ding *et al.* indicated that STAT1 is an important transcription factor for M1 polarization, and identified that physalin D could inhibit M1 polarization by suppressing STAT1 activation 59. However, the role of STAT1 in upregulated M1 polarization in RSA and the underlying mechanism are still largely unknown. In our study, the level of STAT1 and p-STAT1 increased markedly and was negatively related to the expression of miR-103 in RSA patients. Therefore, we hypothesized that decreased miR-103 might be involved in RSA by promoting M1 polarization via disinhibiting STAT1 signal pathway. As expected, our results showed that overexpression of miR-103 led to a decrease in STAT1 by directly interacts with its 3'UTR and subsequently reduced IRF1 expression, while miR-103 knockdown exhibited an opposite result. Moreover, overexpressed STAT1 reversed the inhibitory effects of miR-103 on STAT1/IRF1, and the inhibitory effect of miR-103 on M1 macrophages polarization was also eliminated. All these findings indicated that miR-103 negatively regulates M1 polarization via STAT1/IRF1 signaling pathway. Our study *in vivo* with mice further demonstrated that decreased miR-103 was involved in RSA by promoting M1 polarization via disinhibiting STAT1/IRF1. Overexpressed miR-103 could alleviate embryo loss effectively by suppressing M1 polarization via inhibiting STAT1/IRF1.

In summary, our study revealed that miR-103 is a pivotal regulator of maternal-fetal tolerance by influencing M1 polarization. miR-103 affects the polarization of M1 through the STAT1/IRF1 signaling pathway. miR-103 downregulated STAT1 expression by directly targeting its mRNA 3'UTR and then reduced the expression of IRF1. These findings suggested that the aberrant downregulation of miR-103 contributes to RSA by enhancing M1 macrophage polarization via disinhibiting STAT1/IRF1 signaling pathway. miR-103 might be a potential diagnostic marker and promising therapeutic target for RSA. Meanwhile, it should be noticed that the underlying mechanisms of miRNA regulated RSA are diverse, which might involve various target genes and binding sites. Besides, the interaction network and cross-talk among miRNAs in RSA regulation remain to be fully elucidated in the future study.

## Supplementary Material

Supplementary figures and tables.

## Figures and Tables

**Table 1:** Clinical characteristics of subjects

Subject	RSA (mean± SEM, n= 30)	Control (mean± SEM, n= 30)	*P*
Age (years)	34.00± 0.87	33.43± 1.02	0.67
Number of miscarriages	3.47± 0.11	0	< 0.0001
Gestation age (weeks)	8.36± 0.35	8.72± 0.19	0.36

## Abbreviations

RSA: recurrent spontaneous abortion
NP: normal pregnancy
TNF-α: tumor necrosis factor-α
PM: peritoneal macrophage
M1: classically activated macrophage
M2: alternatively activated macrophage
miRNAs: microRNAs
miR-103: microRNA-103
PBMC: peripheral blood mononuclear cells
NC: negative control
TLR: toll-like receptor ligands
LPS: lipopolysaccharides
IFNγ: interferon-γ
STAT1: signal transducers and activators of transcription 1
IRF1: interferon regulatory factor 1
PPARγ: peroxisome proliferator-activated receptor
WIG1: zinc finger matrin-type 3
KLF6: kruppel like factor 6
C/EBPα: CCAAT enhancer binding protein alpha
GM-CSF: granulocyte-macrophage colony-stimulating factor
EDTA: ethylene diamine tetraacetic acid
IL-6: interleukin-6
IL-12b: interleukin-12b
CCR7: C-C chemokine receptor type 7
CCL2: chemokine (C-C motif) ligand 2
CCL5: chemokine (C-C motif) ligand 5
CCL13: chemokine (C-C motif) ligand 13
CXCL9: chemokine (C-X-C motif) ligand 9
CXCL10: chemokine (C-X-C motif) ligand 10
iNOS: inducible nitric oxide synthase
TNF-α: tumor necrosis factor alpha
CD80: cluster of differentiation 80
CD86: cluster of differentiation 86
MHCII: major histocompatibility complex II
F4/80: mouse EGF-like module-containing mucin-like hormone receptor-like 1
CD14: cluster of differentiation 14
PPARγ: peroxisome proliferator-activated receptor
PARP14: poly (ADP-ribose) polymerase family member 14
qRT-PCR: quantitative real-time polymerase chain reaction
3'UTR: 3' untranslated region
WT: wild-type
MUT: mutant
IHC: Immunohistochemical
FISH: Fluorescence *In Situ* Hybridization
DAPI: 4,6-diamidino-2-phenylindole
ROC: receiver operating characteristic
SD: standard deviation
WHO: World Health Organization
