# Supplementary material for: MiR-103 protects from recurrent spontaneous abortion via inhibiting STAT1 mediated M1 macrophage polarization
Source: Int J Biol Sci. 2020 May 25;16(12):2248–64. doi: 10.7150/ijbs.46144 (PMC7294935; doi:10.7150/ijbs.46144)
Supplement: Supplementary file 1 — Supplementary figures and tables. [file ijbsv16p2248s1.pdf]

Supplementary Figure Legends

Figure S1. M2 macrophages were decreased in RSA patients.

The proportion of $CD14^+$ $CD206^+$ (M2 macrophages) in decidua tissues of NP (n= 30) and RSA case (n= 30). Values were listed as the mean ± SEM. **** *P*< 0.0001.

Figure S2. Expression of 9 predicted miRNAs in decidua tissues.

(A-I) qRT-PCR analysis for expression of miR-23a-3p, miR-23b-3p, miR-20a-5p, miR-20b-5p, miR-106b-5p, miR-17-3p, miR-18a-5p, miR-93-5p and miR-378b in decidua tissues of NP (n= 30) and RSA case (n= 30). Values were listed as the mean ± SEM. * *P*< 0.05, ** *P*< 0.01, *** *P*< 0.001, ns means no statistical difference.

Figure S3. Expression of miR-103 in RAW264.7 and PM cells transfected with miR-103 mimics or miR-103 inhibitor.

(A-B) Expression of miR-103 in RAW264.7 transfected with miR-103 mimics/NC or miR-103 inhibitor/INC for 24 h. (C-D) Expression of miR-103 in PM cells transfected with miR-103 mimics/NC or miR-103 inhibitor/INC for 24 h. Values were listed as the mean ± SEM. ** *P*< 0.01, *** *P*< 0.001, **** *P*< 0.0001.

Figure S4. miR-103 had no effect on IRF5 and IRF8 expression.

RAW264.7 cells were transfected with miR-103 mimics/NC or miR-103 inhibitor/INC, after 24 h, the cells were stimulated with LPS/IFNγ for 24 h. (A) The mRNA level of *IRF5* and *IRF8* were detected in RAW264.7 transfected with miR-103 mimics/NC by qRT-PCR. (B) The protein level of IRF5 and IRF8 were detected in RAW264.7 transfected with miR-103 mimics/NC. (C) The mRNA level of *IRF5* and *IRF8* were detected in RAW264.7 transfected with miR-103 inhibitor/INC by qRT-PCR. (D) The protein level of STAT1 and IRF1 were measured in RAW264.7 transfected with miR-103 inhibitor/INC by western blot. Values were listed as the mean ± SEM. **P*< 0.05, ** *P*< 0.01, ns means no statistical difference.

Figure S1

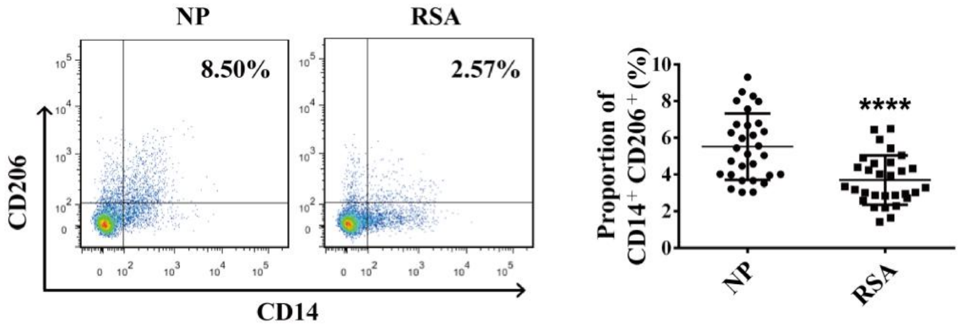

Figure S2

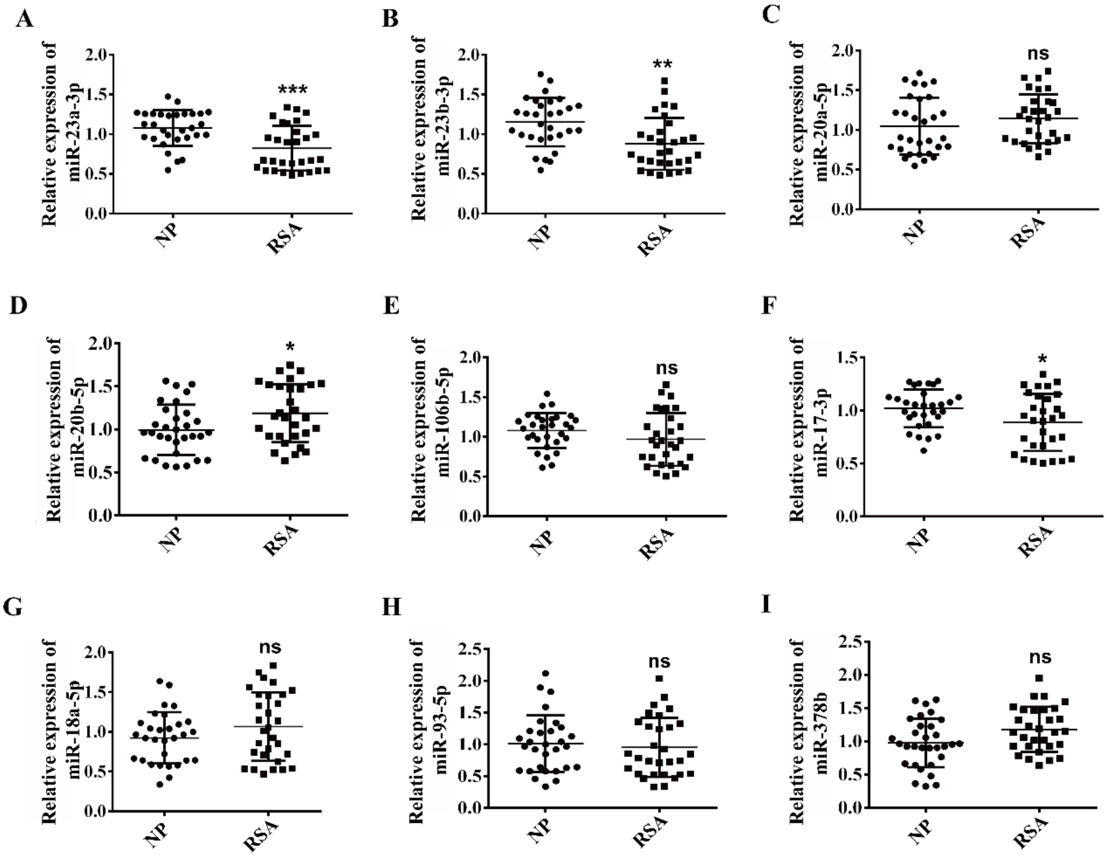

Figure S3

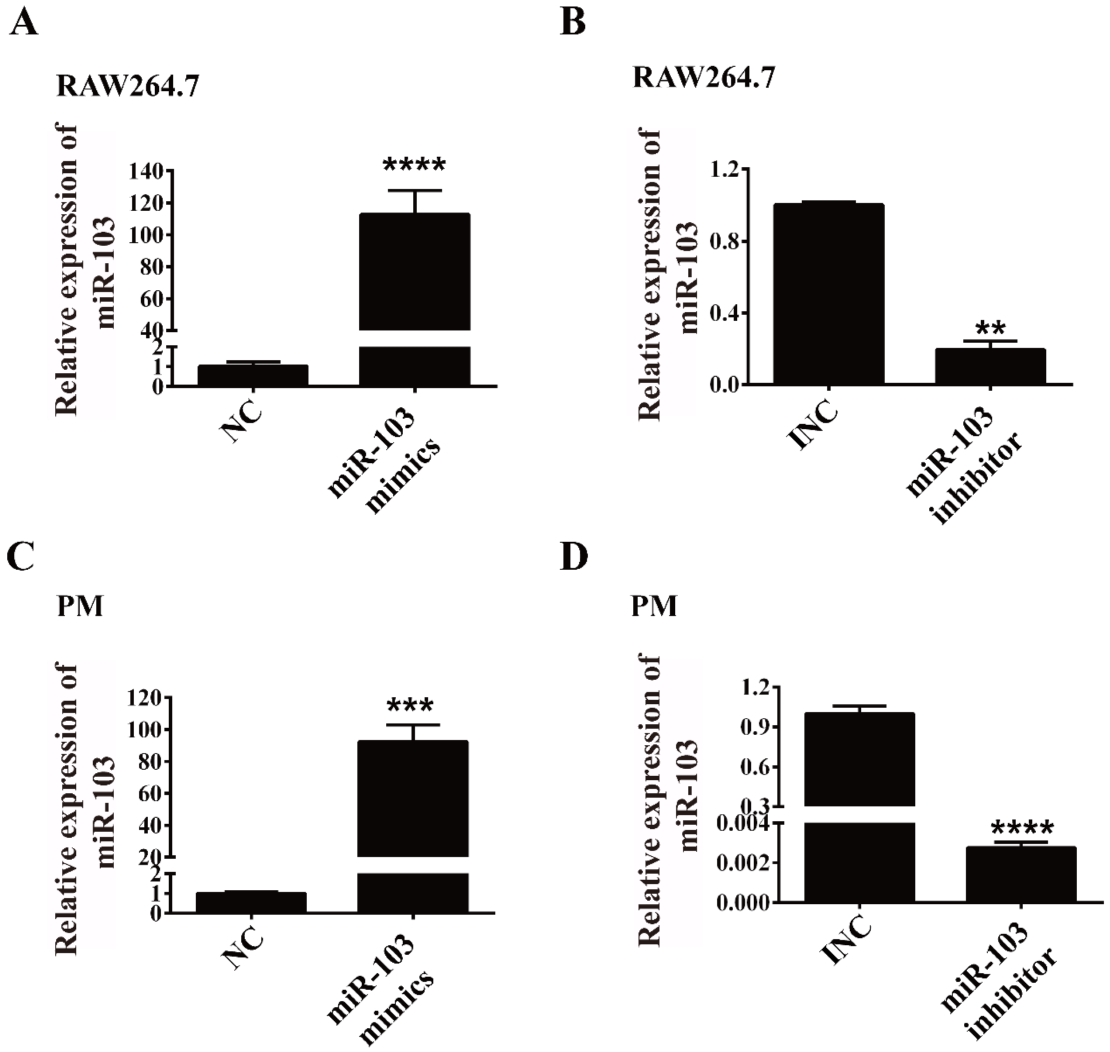
A
RAW264.7
Relative expression of miR-103
140
120
100
80
60
40
2
1
0
****
NC
miR-103 mimics
B
RAW264.7
Relative expression of miR-103
1.2
0.8
0.4
0.0
**
INC
miR-103 inhibitor
C
PM
Relative expression of miR-103
120
100
80
60
40
20
2
1
0
***
NC
miR-103 mimics
D
PM
Relative expression of miR-103
1.2
0.9
0.6
0.3
0.004
0.002
0.000
****
INC
miR-103 inhibitor

Figure S4

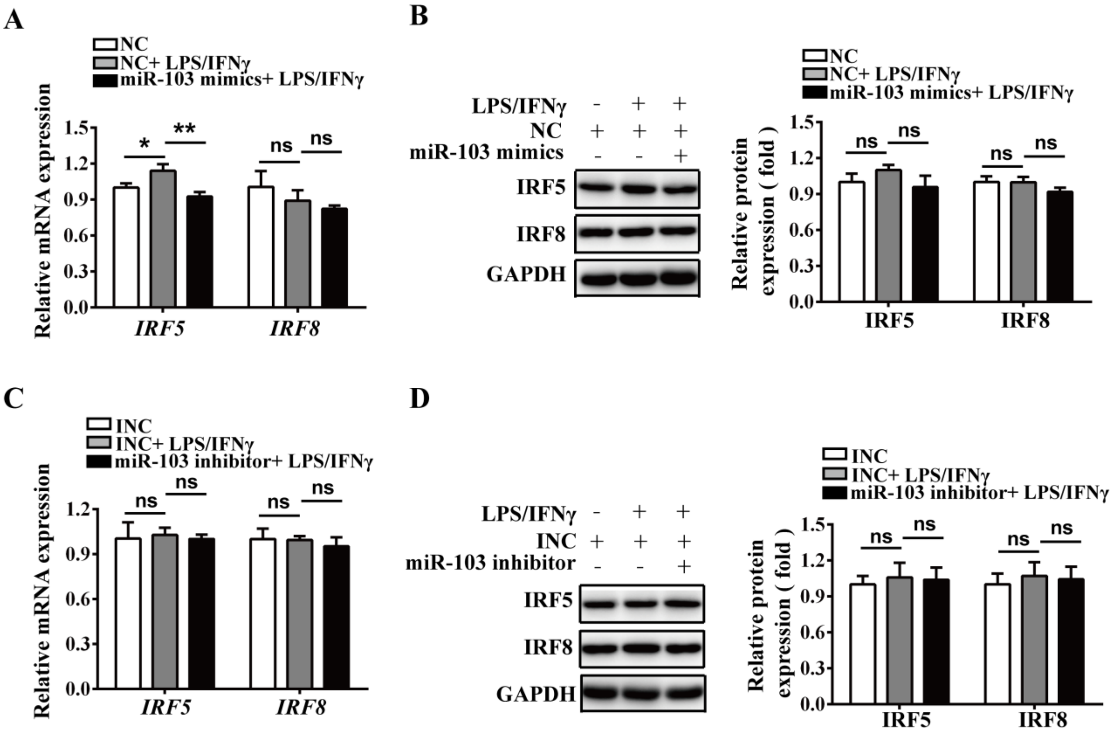

Supplementary Tables

Table S1. miRNAs sequences.

**miRNAs**	**Sequences (5’-3’)**
NC	Sense：UUCUCCGAACGUGUCACGUTT<br>Antisense: ACGUGACACGUUCGGAGAAGAATT
miR-103 mimics	Sense：AGCAGCAUUGUACAGGGCUCUAUGA<br>Antisense：AUAGCCCUGUACAAUGCUGCUUU
INC	CAGUACUUUUGUGUAGUACAA
miR-103 inhibitor	UCAUAGCCCUGUACAAUGCUGCU

Abbreviations: NC, negative control; INC, inhibitor NC.

Table S2. List of primers used in the study.

Gene	Forward primer (5'- 3')	Reverse primer (5'- 3')
**Mus musculus**		
CCL2	TACAAGAGGATCACCAGCAGC	CATTCCTTCTTGGGGTCAGCA
CCL5	TGCCCACGTCAAGGAGTATT	GCGGTTCCTTCGAGTGACA
CXCL9	CCGAGGCACGATCCACTACA	CCGGATCTAGGCAGGTTTGA
CXCL10	GATGACGGGCCAGTGAGAAT	ATCTCAACACGTGGGCAGG
IL6	GGATACCACTCCCAACAGACC	TTCTGCAAGTGCATCATCGT
IL12b	TGGAATGGCGTCTCTGTCTG	GCTTCCAACGCCAGTTCAAT
iNOS	AGCCAAGCCCTCACCTACTT	TCTGCCTATCCGTCTCGTCC
TNF-α	ACGGCATGGATCTCAAAGAC	AGATAGCAAATCGGCTGACG
STAT1	GTTCCGACACCTGCAACTGAA	AGAGGTGGTCTGAAAGGGAAC
IRF1	AAAGTCCAAGTCCAGCCGAG	GTCCGGGCTAACATCTCCAC
IRF5	ATGTTGCCTTTGACGGACCT	TGCTTGTCACTGGGGATGTC
IRF8	TTCTGGTGCAGGTAGAGCAG	AAGGGTCTCTGGTGTGAGGT
ACTB	TCCTTCTTGGGTATGGAATCCTG	TGCTAGGAGCCAGAGCAGTA
**Homo sapiens**		
STAT1	TAATCAGGCTCAGTCGGGGA	CCACACCATTGGTCTCGTGT
ACTB	GGGAAATCGTGCGTGACATT	GGAACCGCTCATTGCCAAT
**miRNAs**		
miR-103	CAGATAGCAGCATTGTACAGGG	TATCGTTGTACTCCAGACCAAGAC
miR-23a-3p	GAAGTCTATCACATTGCCAGGG	TATGGTTGTTCTCGTCTCTGTGTC
miR-23b-3p	TGGGTTCCTGGCATGCTGATTT	GTCGTATCCAGTGCGTGTCGTG
miR-20a-5p	GCCCGCTAAAGTGCTTATAGTG	GCTGTCAACGATACGCTACGT
miR-20b-5p	CAAAGTGATCATAGTGCAGGTA	GGGACCTTGGTTAGGTGCAC
miR-106b-5p	TGCGGCAACACCAGTCGATGG	CCAGTGCAGGGTCCGAGGT
miR-17-3p	CTCAACTGGTGTCGTGGA	ACTTGTAGCTCAACT
miR-18a-5p	ACGTAAGGTGCATCTAGTGVAGAT	GTGCAGGGTCCGAGGT
miR-93-5p	GCCATGTAAACATCTCGGACTG	CAATGCGTGTGGTGGAGGAG
miR-378b	GGTCATTGAGTCTTCAAGG	GGTCTTTCTGCCTCCA
U6	ATTGGAACGATACAGAGAAGATT	GGAACGCTTCACGAATTTG

Abbreviations: *CCL2*, C-C motif chemokine ligand 2; *CCL5*, C-C motif chemokine ligand 5; *CXCL9*, C-X-C motif chemokine ligand 9; *CXCL10*, C-X-C motif chemokine ligand 10; *IL6*, interleukin 6; *IL12b*, interleukin 12b; *iNOS*, inducible NO synthase; *TNF-α*, tumor necrosis factor-α; *STAT1*, signal transducer and activator of transcription 1; *IRF1*, interferon regulatory factor 1; *IRF5*, interferon regulatory factor 5; *IRF8*, interferon regulatory factor 8; *ACTB*, beta-actin.

Table S3. Differentially expressed miRNAs in RAW264.7-derived M1 macrophage microarray.

miRNA	*P* value	Fold change	Regulation
miR-21a-3p	4.14E-05	105.6048	up
miR-146b-5p	0.015174	17.5164	up
miR-7011-3p	0.037886	13.36382	up
miR-5121	0.039008	8.273133	up
miR-125a-5p	8.69E-05	7.953795	up
miR-22-3p	2.08E-04	7.754936	up
miR-99b-5p	0.014278	6.597976	up
miR-146a-5p	1.81E-05	3.953452	up
let-7e-5p	2.20E-05	3.264867	up
miR-210-3p	0.019694	2.911234	up
miR-222-3p	0.001151	2.792338	up
miR-6931-5p	0.002041	2.543805	up
miR-466h-3p	0.027037	2.390977	up
miR-29a-3p	0.001455	2.308446	up
miR-1224-5p	7.54E-04	2.281455	up
miR-8110	0.003366	2.200346	up
miR-466f-3p	0.044144	2.173257	up
miR-6769b-5p	0.018995	2.131759	up
miR-466q	0.044854	2.117526	up
miR-574-5p	0.016038	2.097379	up
miR-1897-5p	0.001047	2.082543	up
miR-378a-3p	0.007884	33.20296	down
miR-378b-3p	0.007699	23.11563	down
miR-301a-3p	0.045478	18.83452	down
miR-20b-5p	9.00E-04	5.628574	down
miR-93-5p	2.48E-04	4.998238	down
miR-20a-5p	7.72E-07	4.912466	down
miR-18a-5p	0.002095	4.820261	down
miR-425-5p	0.005142	4.343293	down
miR-92a-3p	9.28E-06	3.848692	down
miR-15b-5p	2.92E-05	3.842767	down
miR-27b-3p	1.55E-04	3.763017	down
miR-30c-5p	1.45E-05	3.697379	down
miR-25-3p	1.41E-04	3.488334	down
miR-19a-3p	0.001285	3.295047	down
miR-484	0.003361	3.174037	down
miR-423-5p	3.08E-04	3.08423	down
miR-19b-3p	7.69E-04	3.03592	down
miR-193a-3p	0.013033	3.020537	down
miR-106b-5p	0.006581	2.939195	down
miR-17-3p	9.08E-04	2.891409	down
miR-103-3p	0.014943	2.888302	down
miR-130b-3p	0.002611	2.811731	down
miR-223-3p	0.002205	2.637563	down
miR-27a-3p	0.001053	2.633079	down
miR-30a-5p	0.004397	2.6166	down

let-7c-5p	0.002998	2.487104	down
miR-1904	1.06E-04	2.450692	down
miR-23b-3p	0.00381	2.333629	down
miR-99a-5p	0.001088	2.312916	down
miR-23a-3p	7.36E-05	2.099742	down
